# Utilization of long-acting contraceptive methods and associated factor among women of reproductive age in East Africa: A multi-level analysis of recent Demographic and Health Surveys

**DOI:** 10.1371/journal.pone.0319003

**Published:** 2025-03-12

**Authors:** Habtu Kifle Negash, Ayenew Molla Lakew, Gebretsadik Endeshaw Molla, Adhanom Gebreegziabher Baraki, Yitbarek Fantahun Mariye, Winta Tesfaye, Bezawit Habtamu Bekele, Biruk Lelisa Eticha

**Affiliations:** 1 Department of Anatomy, College of Medicine and Health Sciences, University of Gondar, Gondar, Ethiopia; 2 Department of Epidemiology and Biostatistics, Institute of Public Health, College of Medicine and Health Sciences, University of Gondar, Gondar, Ethiopia; 3 Ayera General Hospital, Gondar, Ethiopia; 4 Queen’s University, School of Rehabilitation Therapy, Kingston, Ontario, Canada; 5 Department of Obstetrics & Gynecology, School of Medicine, College of Medicine & Health Sciences, Addis Ababa University, Addis Ababa, Ethiopia; 6 Department of Physiology, College of Medicine and Health Sciences, University of Gondar, Gondar, Ethiopia; 7 Department of Epidemiology and Biostatistics, Institute of Public Health, College of Health Sciences, Addis Ababa University, Addis Ababa, Ethiopia; 8 Department of Optometry, School of Medicine, University of Gondar, Comprehensive Specialized Hospital, Gondar, Ethiopia; University of Salamanca, SPAIN

## Abstract

**Background:**

Comprehensive family planning is essential for reproductive health, allowing individuals to make informed choices about family size and enhancing maternal and child health. Long-acting contraceptives (LACs) are known for their high efficacy and consistent use. This study examines the prevalence and determinants of LAC utilization among women of reproductive-age in 11 East African countries.

**Methods:**

Secondary data from Demographic and Health Surveys (2015-2022) across 11 East African nations were pooled and analyzed. The study sample included 144,414 women aged 15–49. Bivariate and multivariate regression analyses were conducted using Stata 17 to explore factors associated with LAC utilization. Results are presented as adjusted odds ratios (AOR), with statistical significance at p <  0.05.

**Results:**

The prevalence of LAC utilization among reproductive-age women in East Africa is 14.87%. Women from middle-income households are 13% more likely to utilize LACs compared to those from poor households (AOR = 1.13, 95% CI: 1.08–1.18), while those from wealthy households are 15% more likely (AOR = 1.15, 95% CI: 1.11–1.20). Married women and those living with a partner are 42% more likely to utilize LACs than unmarried women (AOR = 1.42, 95% CI: 1.33–1.52). Educational attainment significantly impacts LAC utilization: women with primary education are 52% more likely (AOR = 1.52, 95% CI: 1.44–1.60), those with secondary education are 63% more likely (AOR = 1.63, 95% CI: 1.53–1.73), and women with higher education are twice as likely to utilize LACs (AOR = 2.00, 95% CI: 1.84–2.17).

**Conclusions:**

LAC utilization remains relatively low in the studied East African countries. Factors positively associated with higher LAC utilization include being married, higher income, educational attainment, employment, and media exposure. Additionally, women with more than three children and those from countries with lower illiteracy and poverty rates also have higher LAC utilization. Conversely, a history of abortion, fewer health fieldworker visits, and later initiation of sexual activity and childbirth are associated with lower LAC utilization. These findings emphasize the need for targeted interventions to overcome barriers and promote LAC access and utilization.

## Introduction

According to the World Health Organization, achieving the ideal family size and making educated decisions about one’s own reproductive health are made possible by comprehensive family planning, which also improves the health of mothers and children [1,2].

As a family planning strategy, contraceptives fall into two categories: long-acting (reversible or permanent) methods, such as implants, intrauterine devices (IUDs), and sterilization; and short-acting methods, including, injectables, pills, condoms, spermicides, and other traditional techniques [3,4]. Long-acting contraceptives (LACs) are now one of the safest, most successful, and efficient family planning techniques available [5,6], providing protection against pregnancy for at least three years [3].Unchecked and unplanned rapid population growth remains a significant challenge to social, economic, and political stability in Africa, particularly in the sub-Saharan region (SSA), where fertility rates there range from 2.9% to 7.2%. This growth has led to environmental degradation, poverty, and poor family health, among other serious issues [7]. Globally, approximately 64% of fertile women utilize family planning methods, with LACs predominantly utilized in Asia and North America, accounting for 34% of this total. In contrast, the utilization rate of LACs in SSA remains low, at only 5.1% [8]. While LAC utilization in sub-Saharan Africa started at very low levels, it has experienced significant double-digit growth over the past decade [5]. Contraceptive utilization in East Africa has also increased significantly increased [9]. Between 2000 and 2004, the utilization rate was 24%, rising to 39% between 2015 and 2019. Specifically, utilization increased significantly across several countries: Ethiopia from 8% to 36%, Kenya from 39% to 58%, Rwanda from 17% to 53%, Tanzania from 25% to 34%, and Uganda from 23% to 39%. This rise also includes an increase in long-acting contraceptives, though less pronounced than short-term methods. Between 2014 and 2016, long-acting contraceptive utilization was 10% in Ethiopia, 17% in Kenya, 10% in Rwanda, 11% in Tanzania, and 11% in Uganda up from much lower levels in previous years [10].Factors significantly correlated with the utilization of LAC include a women’s age [11], marital status, employment status [12], education level [5], wealth [13], place of residence [14], history of pregnancy termination [15], total number of living children [16], and fertility preferences [17].

Despite numerous studies on LAC, there is limited evidence that comprehensively addresses both community and individual level factors across broader study populations. This study is among the limited multi-level regional analyses aimed at understanding the actual practice of LAC utilization in East Africa: a sub-Sahara region suffering with poverty driven by uncontrolled fertility and high mortality rates.

This study aims to fill critical knowledge gaps regarding the prevalence and determinants of LAC utilization among reproductive-age women in East Africa by analyzing recent Demographic and Health Surveys (DHS). It provides new insights into how socio-economic factors, educational attainment, and marital status influence LAC access and utilization. Moreover, the evidence generated could guide the development of effective programs and strategies to tackle uncontrolled fertility-related challenges. By promoting LAC, a reliable, efficient, and widely respected family planning method, this study seeks to empower grassroots efforts to mitigate these issues.

## Methods

### Study design, setting and period

**Fig 1:**
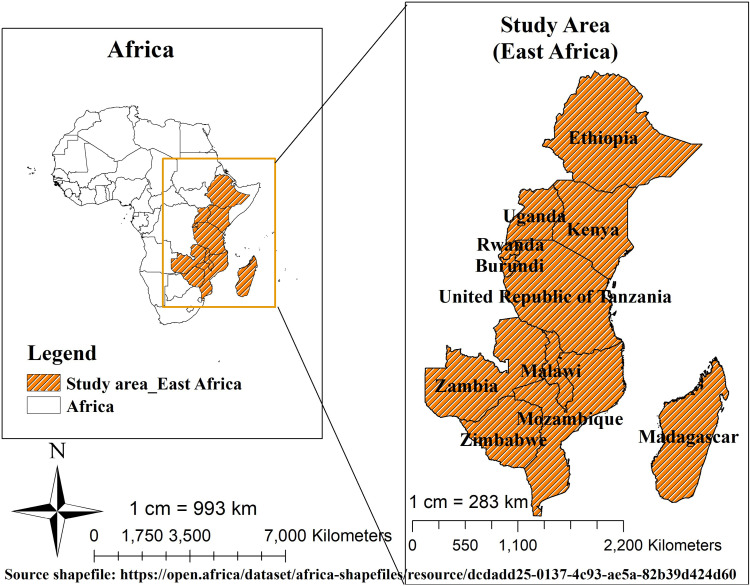
Map of East African countries (19).

This study used cross-sectional data from the Demographic and Health Surveys, which have been conducted since 1984 in over 85 countries. DHS provides comprehensive data on fertility, health, and nutrition, with strong national representation and uniform methodologies, aiding in epidemiological research. [18]. Thus, the current study was based on health and demographic surveys that were carried out in East African nations (Fig 1)[19] between 2015 and 2022.

### Data source and measurements

Data for this study were obtained from the Demographic and Health Surveys conducted in eleven East African countries: Burundi, Ethiopia, Kenya, Madagascar, Malawi, Mozambique, Rwanda, Tanzania, Uganda, Zambia, and Zimbabwe. Unfortunately, recent DHS data for Sudan, Eritrea, and Comoros were unavailable at the time of our analysis. DHS surveys, carried out every five years, use validated instruments and standardized methods to ensure consistency across low- and middle-income countries. The DHS program oversees global data collection and coding, utilizing uniform sampling techniques and questionnaires, which facilitates reliable multi-country comparisons [20].

The DHS employs a stratified two-stage sampling process to ensure national representation. Initially, enumeration areas (EAs) are randomly selected from a sampling frame based on the latest available national census. In the second stage, households within these EAs are systematically chosen, focusing on families with women aged 15 to 49. This study included women (aged 15-49) in this demographic who had sought information on contraception. The final dataset comprised 144,414 respondents, including 7,780 from Zimbabwe and 25,783 from Kenya (Table 1).

**Table 1:** The study participants by country and respective year of the survey.

Country	Year of survey	Unweighted frequency (n)	Percent (%)	Weighted frequency (n)
Burundi	2016/2017	10,601	7.34	10,865.08
Ethiopia	2016	10,071	6.97	10,378.37
Kenya	2022	25,783	17.85	26,269.04
Madagascar	2021	14,814	10.26	14,806.82
Malawi	2015/2016	19,899	13.78	20,048.43
Mozambique	2015	10,066	6.97	10,028.29
Rwanda	2019/2020	9,481	6.57	9,582.81
Tanzania	2022	11,081	7.67	11,513.74
Uganda	2016	14,236	9.86	14,208.23
Zambia	2018	10,602	7.34	10,634.19
Zimbabwe	2015	7,780	5.39	77,58.93
Total		144,414	100	146,093.93

### Dependent variable

The utilization of long-acting contraceptives was the study’s dependent variable. The DHS databases included information on women of reproductive-age who inquired about the forms of contraception that were currently in use. Menopause, infertile women, and women who had never had intercourse were not included. In order to fit the multilevel binary logistic regression model, women who used one of the long-acting methods of contraception; IUDs, implants, or female sterilization, were reclassified as LAC utilizers (coded as “1”) and non-LAC utilizers (coded as “0”) [15].

### Independent variables

The study examines individual-level factors from the most recent DHS datasets, including maternal age (15–24, 25–34, 35–49), the mother’s educational background (no formal education, primary, secondary, higher education), her employment status (working, not working), her marital status (never married, married & living with partner, widowed, divorced & separated), the age at first sexual activity (<18, ≥ 18), the number of living children (1–2, 3–4, > 4 children), her wealth status (poor, middle, rich), her history of terminated pregnancy (yes, no), her perception of the distance to the health facility (big problem, not a big problem), the age at first birth (≤20, > 20), media exposure (yes, no), number of visits to health facilities or health extension workers in the last year (yes, no), and the source of contraception (government, non-governmental organization, private, unknown).

On the other hand, community-level characteristics included dwelling type (rural or urban), poverty level (low, high), women’s education level (low, high), and media exposure (low, high).

By combining individual-level characteristics at the national level, the aggregate community-level explanatory variables (community poverty level, community illiteracy level, and community media exposure) were calculated. Following the use of the histogram to verify the distribution, they were classified as high or low depending on the distribution of the proportion values generated for each community (Table 2). The regularly distributed median value served as the categorization cut-off point if the aggregate variable had a normally distributed mean value, and the normally distributed median value if it did not.

**Table 2:** Definitions and Operationalization of Variables.

Variables	Measurements/definitions
Long acting contraceptive utilizers	Women who were used one of the long acting contraceptive methods: IUD, Implant and Female sterilization considered as LACs utilizer [15]
Community level media Exposure	At the cluster level, clusters with a population above the median who were exposed to family planning messages in the media were classified as having “high media exposure” [21].
Community level illiteracy	At the cluster level, clusters with a population above the median who did not receive any form of education were classified as having a “high illiteracy level” [21].
Community level poverty	Clusters were evaluated based on aggregated poverty measures. Those with poverty levels above the median and categorized as “poor” or “poorest” were classified as having a “high poverty level” [21].
Large Family	A family is defined as “large” if it has four or more children [22].

## Operational definitions

### Statistical analysis

STATA version 17 was used for data extraction, re-coding, and analytical and descriptive analysis. In order to make the sample as representative as possible and make the entire sample resemble the population of the nation, weighting was used. Cross-tabulation was used for descriptive analysis. In order to illustrate the percentages of LAC utilizers and their distribution throughout a few chosen nations, the frequency of certain features was displayed using pie and bar charts. The hierarchical structure of the data from the demographic health survey made the multilevel analysis suitable. Since the dependent variable in this study was binary, the multilevel mixed-effects generalized linear model was used. First, variables were chosen for multi-variable analysis using a bi-variable analysis; variables that showed a p-value of less than 0.20 in the bi-variable analysis may be included in the multi-variable analysis. Four models were fitted after the candidate variables were chosen for the bi-variable analysis: the null model, which included no variables; model II, which was adjusted for variables at the individual level only; model III, which was adjusted for variables at the community level only; and model IV, which was adjusted for both individual and community level variables at the same time. The settings for the fixed effect AOR with 95% Confidence Interval and a p-value less than 0.05 were utilized to indicate statistical significance.

The deviance and log-likelihood ratio tests were used to compare models prior to interpreting multilevel logistic regression. The intra-class correlation coefficient (ICC) $ICC=\frac{VC}{VC+3.29}\times 100\%$, proportionate change in variance (PCV); $PCV=\frac{Vnull-VC}{Vnull}\times 100\%$, and median odds ratio (MOR) were used to quantify the random effects, or fluctuation of effects. The ICC explains the cluster variability [23], while PCV can measure the total variation due to factors at the community and individual level; MOR reflects the unexplained cluster heterogeneity and measures the area variance as odds ratios [24,25]. The models with the lowest deviance, the highest PCV, and the lowest MOR; $MOR=e{}^{{}^{0.95\surd VC}}$ were selected as the better-fitted models for interpretation [26,27]. A multi-collinearity test was done to rule out a significant correlation between independent variables.

### Ethical approval and consent to participate

Ethical approval for this study was not required because it utilized existing public domain survey datasets, which are freely available at www.measuredhs.com, with all identifying information removed. However, we obtained permission and approval from Measure DHS through their online request process to access and use the data.

### Result

#### Socio-demographic characteristics of study participants

The study included 146,094 participants from eleven East African nations, 72.36% were married and living with their partner, while 85.98% reported no history of pregnancy termination. For more than half of respondents (65.62%) distance from the health facility was perceived as not a big problem. Additionally, less than one-fifth (14.16%) of respondents had received at least one visit by healthcare fieldworkers in the past year (Table 3).

**Table 3:** Individual and household level characteristics of respondents in Eastern Africa.

Variables	Category	Weighted Frequency (n)	Percent (%)
Age in years			
	15-24	46,653	31.93
	25-34	55,369	37.90
	35-49	44,071	30.17
Marital status			
	Never married	21,806.27	14.93
	Married and living with a partner	105,717.67	72.36
	Widowed, divorced, or separated	18,570	12.71
Wealth index			
	Poor	53,736.56	36.78
	Middle	27,545.46	18.85
	Rich	64,811.91	44.36
Educational status			
	No education	25,570.94	17.5
	Primary	68,978.2	47.21
	Secondary	40,745.4	27.89
	Higher	10,799.4	7.39
Working status			
	Not working	53,853.1	36.86
	Working	92,240.83	63.14
Terminated pregnancy			
	No	125,615.02	85.98
	Yes	20,478.91	14.02
Visited by HFW in the last 12 months			
	No	125,411.79	85.84
	Yes	20,682.14	14.16
Visited HF in the last 12 months			
	No	47,755.33	35.89
	Yes	85,295.2	64.11
Age at 1^st^ sex			
	<18	87,206.94	59.69
	≥18	58,886.99	40.31
Age at 1^st^ birth			
	≤20	86,160.127	58.98
	>20	59,933.8043	41.02
Source of contraceptive			
	Government	45,448.47	31.11
	NGO	1,920.43	1.31
	Private	24,194.83	16.56
	Do not know	74,530.2	51.02
Distance from health facility			
	Big problem	50,227.879	34.38
	Not big problem	95,866.05	65.62
Media exposure			
	No	45,559.25	31.18
	Yes	100,534.68	68.82
Number of living children			
	No children	20,724.17	14.19
	1-2 Children	55,893.78	38.26
	3-4 Children	40,228.52	27.54
	≥5 children	29,247.46	20.02
Desire for more children			
	Want other	76,186.56	52.15
	Undecided	6,926.56	4.74
	Want no more	62,980.81	43.11

Around three-fourths of the respondents resided in rural areas. The above half of the study subjects got low-level education and media exposure. 48.24% of participants were living in households with high levels of poverty (Table 4).

**Table 4:** Community-level characteristics of respondents in Eastern Africa.

Variables	Category	Weighted Frequency (n)	Percent (%)
Residence			
	Urban	41,875.43	28.66
	Rural	104,218.5	71.34
Community illiteracy			
	Low	72,125.955	49.37
	High	73,967.976	50.63
Community media exposure			
	Low	78,382.826	53.65
	High	67,711.105	46.35
Community poverty level			
	Low	75,624.085	51.76
	High	70,469.8462	48.24

#### Random effect and model comparison

(Table 5) detailed the random effects and country-level variation in LAC utilization, along with a comparison of different models. Initially, a null model was used to perform variance component analysis to break down the overall variance in LAC utilizers. In this model, the country was used as a level-two variable. The analysis estimated the country-level variance, reflecting the proportion of total variance in LAC utilization attributable to the specific country context in which the women resided. The significance of this country-level variance was confirmed by the statistical results [country variance =  1.12; standard error (SE) =  0.48; P-value =  0.001], indicating significant differences in LAC utilization between countries. This was further evidenced by the intraclass correlation coefficient in the null model, which showed that approximately 25.4% of the variation in LAC utilization among women could be explained by country-level factors.

**Table 5:** Random effect and model fitness test result in the adjusted multilevel regression model.

Random effect result	Null model (I)	Model II	Model III	Model IV
Community (country) variance (SE)	1.12 (0.48)	1.06 (0.46)	1.1 (0.48)	1.05 (0.45)
ICC %	25.4 (0.13, 0.44)	24.34(0.12, 0.43)	25.14 (0.13, 0.44)	24.2 (0.12, 0.43)
MOR	2.9	1.54	2.86	1.54
PCV%	**Reference**	5.52	1.4	6.23
AIC	112923.2	105566.9	112682.7	105451.6
BIC	112943	105804	112742	105728.2
Deviance	112,919.22	105518.88	112,670.74	105395.56
Log-likelihood	-56459.61	-52759.44	-56335.37	-52697.78

ICC=Intra-class Correlation Coefficient: MOR: Median Odds Ratio: PCV: Proportional Change in Variance: AIC =  Akaike Information Criterion: BIC = Bayesian Information Criterion

Additionally, the final model (Model IV) revealed that about 24.2% of the variation in LAC utilization was attributed to both individual-level and country-level factors. To evaluate model fit, we used AIC, BIC, and deviance metrics. Model IV, which had the lowest values for AIC, BIC, and deviance, was identified as the best-fitting model (see Table 5).

The analysis also found a mean Variance Inflation Factor (VIF) of 1.7, indicating no significant collinearity among the variables. (Table 5)

#### Long-acting contraceptive utilization

**Fig 2:**
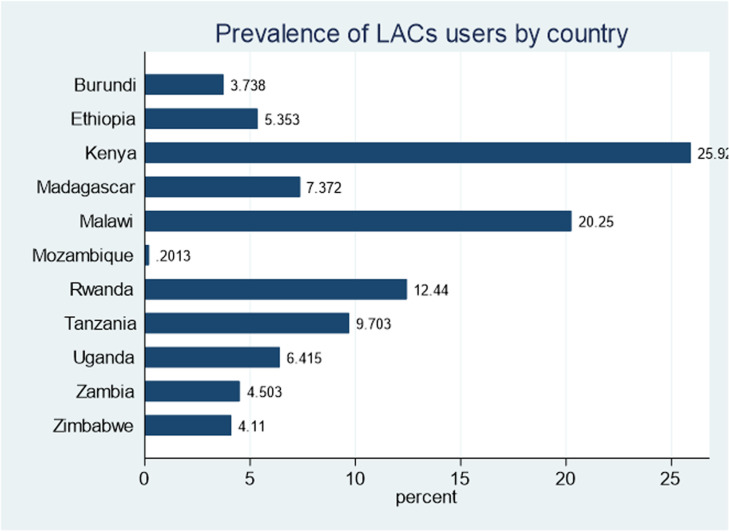
Prevalence of long-acting contraceptives use among East African nations.

The proportion of LAC utilization among reproductive-age women from East African countries was found to be 14.87% (95% CI: 0.147, 0.151). Kenya (25.92%), Malawi (20.25%), and Rwanda (12.44%) were the top three countries that had a large proportion of LAC utilization among reproductive-age women (Fig 2).

#### Associated factors of long-acting contraceptives utilization

After controlling for other individual and community level factors, the odds of LAC utilization among participants living in middle and rich households were increased by 13% (AOR = 1.13, 95% CI:1.08, 1.18) and 15% (AOR = 1.15, 95% CI:1.11, 1.2) respectively as compared to poor participants. Regarding maternal marital status, married & women who live with a partner had a 42% (AOR = 1.42; 95% CI;1.33, 1.52) higher utilization of LACs as compared to unmarried women.

Reproductive-age women who had previously terminated a pregnancy (AOR = 0.85, 95% CI:0.81, 0.89), those who had visited HFWs within the previous year (AOR = 0.90, 95% CI:0.86, 0.94), those who began having sex before the age of 18 (AOR = 0.95, 95% CI:0.91, 0.98), and those who had their first child before the age of 20 (AOR = 0.83, 95% CI:0.80, 0.87) were less likely to utilize LACs than those who had no prior history of terminated pregnancy, those who had a visit from HFWs, those whose first sexual experience occurred before the age of 18, and those who had their first child after the age of 20, respectively.

Women in the reproductive-age group who are employed (AOR = 1.28, 95% CI:1.24, 1.34), those who have been exposed to the media (AOR = 1.21, 95% CI:1.17, 1.26), and women who do not wish to have any more children (AOR = 1.21, 95% CI:1.17, 1.26) are more likely to utilize LACs than are not employed, those who have not been exposed to the media, and those who would like to have more children, respectively.

A higher likelihood of utilizing LACs is often associated with education. When compared to illiterate women, women with primary (AOR = 1.52 [95% CI: 1.44, 1.6]), secondary (AOR = 1.63 [95% CI:1.53, 1.73]), and higher education (AOR = 2.00 [95% CI:1.84, 2.17]) have higher utilization.

When compared to mothers with one to two children, the odds of using LACs increased for those with three to four children by 19% (AOR 1.19, 95% CI: 1.14, 1.24) and for those with more than five children by 28% (AOR 1.28, 95% CI: 1.21, 1.35). On the other hand, compared to women with one to two children, the utilization of LACs was 87% (AOR 0.13, 95% CI: 0.11, 0.14) lower in those without children.

Furthermore, women of reproductive-age have higher odds of using LACs if they come from lower illiteracy countries (13%; AOR = 0.87; 95% CI; 0.8, 0.92), higher poverty countries (9%; AOR = 1.09, 95% CI:1.05, 1.13), and higher media exposure countries (13%; AOR = 1.13, 95% CI:1.09, 1.18) than those from high community illiteracy, low poverty, and low media exposure, respectively (Table 6).

**Table 6:** Multilevel mixed effect logistic regression analysis of individual and community level factors associated with LACs utilization among reproductive-age women in East Africa.

Variables	Model I (null)	Model II AOR (95%CI)	Model III AOR (95%CI)	Model IV AOR (95%CI)
**Age of the respondent (in years)**				
15-24	–	1.00	–	1.00
25-34	**–**	1.2(0.97, 1.07)	–	1.01(0.97, 1.07)
35-49	–	1.00(0.95, 1.07)	–	1.00(0.94, 1.06)
**Marital status**				
Never married	–	1.00	–	1.00
Married and living with a partner	–	1.42(1.33, 1.52)***	–	1.43(1.34, 1.52)***
Widowed divorced and separated	–	1.02(0.94, 1.10)	–	1.02(0.95, 1.11)
**Wealth index**				
Poor	–	1.00	–	1.00
Middle	–	1.13(1.08, 1.18)***	–	1.13(1.08, 1.19)***
Rich	–	1.56(1.11, 1.2)***	–	1.16(1.11, 1.21)***
**Educational status**				
No education	–	1.00	–	1.00
Primary	**–**	1.52(1.44, 1.6)***	–	1.47(1.4, 1.55)***
Secondary	–	1.63(1.53, 1.73)***	–	1.57(1.47, 1.67) ***
Higher	–	2.00(1.84, 2.18)***	–	1.93(1.77, 2.1) ***
**Working status**				
Not working	–	1.00	–	1.00
Working	–	1.29(1.24, 1.34)***	–	1.28(1.24, 1.33) ***
**Terminated pregnancy**				
No	–	1.00	–	1.00
Yes	–	0.85(0.81, 0.89)***	–	0.85(0.81, 0.89) ***
**Visit by HFW in the last 12 months**				
No	–	1.00	–	1.00
Yes	–	0.90(0.86, 0.94)***	–	0.90(0.86, 0.94)***
**Health facility visits in the last 12 months**				
No	–	1.00	–	1.00
Yes	–	0.99(0.95, 1.02)	–	0.99(0.95, 1.02)
**Age at first sex**				
< 18	–	1.00	–	1.00
≥ 18	–	0.94(0.91, 0.98)**	–	0.95(0.91, 0.98) **
**Age at 1**^**st**^ **birth**				
≤ 20	–	1.00	–	1.00
> 20	–	0.83(0.8, 0.87)***	–	0.83(0.80, 0.87)***
**Desire for more children**				
Want other	–	1.00	–	1.00
Undecided	–	0.96(0.89, 1.04)	–	0.96(0.89, 1.05)
Want no more	–	1.58(1.52, 1.64)***	–	1.57(1.51, 1.64) ***
**Distance to a health facility**				
Not big problem	–	1.00	–	1.00
Big problem	–	0.96(0.93, 0.999)*	–	0.97(0.93, 1.00)
**Media exposure**				
No	–	1.00	–	1.00
Yes	–	1.24(1.12, 1.23)***	–	1.21(1.17, 1.26) ***
**Number of Living Children**				
1-2 Children	–	1.00	–	1.00
No children	–	0.13(0.11, 0.14)***	–	0.13(0.11, 0.14) ***
3-4 Children	–	1.19(1.14, 1.24)***	–	1.19(1.14, 1.24) ***
≥ 5 children	–	1.28(1.21, 1.35)***	–	1.28(1.21, 1.35)***
**Residence**				
Urban	–	–	1.00	1.00
Rural	–	–	0.98(0.9, 1.01)	0.99(0.95, 1.03)
**Community illiteracy**				
Low	–	–	1.00	1.00
High	–	–	0.85(0.82, 0.88)***	0.87(0.8, 0.92) ***
**Community media exposure**				
Low	–	–	1.00	1.00
High	–	–	1.18(1.13, 1.22)***	1.13(1.09, 1.18) ***
**Community poverty level**				
Low	–	–	0.11	1.00
High	–	–	1.08(1.05, 1.12)***	1.09(1.05, 1.13)**

N =  sample size, HFW = Health Fieldworkers,

*  =  P value <  0.05,

**=  P value <  0.001,

***=  P value <  0.001.

## Discussions

Modern family planning techniques that are both practical and extremely successful, long-acting contraceptives can prevent pregnancy and significantly reduce expenditures for both governments and couples [28]. This study identified factors that influence the utilization of long-acting contraceptive methods among childbearing-age women in 11 Eastern African countries. The prevalence of LAC utilization ranged from 0.2% in Mozambique to 25.92% in Kenya and overall, the prevalence of LAC utilization in the 11 Eastern African countries was 14.87%. This finding is comparable to those of studies conducted among reproductive-age women in Ethiopia (11.6%, 16%) [29,30], Zimbabwe (15.73%) [17], and Nigeria (14.8%) [31]. The similar social and political environments of the Sub-Saharan African nations may be the cause of the similarities. This result is higher than in a study conducted among women in SSA (DR. Congo, Niger, Angola, Gambia) that reported LACs utilization of < 10% [32]. However, the finding was lower than the studies done in SSA 21.73% [17], Kenya 20.6% [33], Chad 89% [34], and Mali 44.3% [32]. The discrepancy may arise from the diversity of participant characteristics as well as the individual and community-based design of our research. Over time, LAC uptake has notably increased across several Sub-Saharan African regions: in Malawi, it surged from 0.46% in 2004 to 10.21% in 2016; in Zimbabwe, it rose from 1.04% in 2006 to 9.27% in 2016; and in Rwanda, it grew from 0.79% in 2005 to 7.68% in 2015 [5].

The final model indicates that 24.2% of the variation in the prevalence of LAC utilization among women in East Africa who are of reproductive-age is attributed to both individual and community-level factors.

The regression analysis confirmed the presence of multiple individual and community-level factors that had a significant association with LAC utilization. Marital status, wealth index, educational status, working status, history of a terminated pregnancy, visit by community health workers, age at first sex, age at first birth, desire for more children, media exposure, and the number of living children were individual factors captured to have a significant association with LACs use, whereas variables such as community illiteracy, media exposure, and poverty level were the significantly associated community-level factors.

This study confirmed that the odds of LAC utilization among married participants currently living with their partner increased by 43% as compared to single women. This finding was consistent with studies conducted on SSA utilizing DHS data [35], Nigeria [31], Ghana [36], and Ethiopia [37]. This consistency may have arisen because married women who are currently living with their partner have a higher chance of becoming pregnant than single women. Consequently, to prevent unintended pregnancies, delay and space out pregnancies, leaving them with no choice but to embrace LACs methods [38]. However, this contradicts a study conducted in the Democratic Republic of Congo (DRC) that reported that modern methods and LACs utilization were lower among married women [39]. This may be connected to women’s empowerment, husbands being the primary decision-makers, and pressure from family and society to have children soon after marriage.

These contrasting results highlight the complexity of family planning behaviors and underscore the importance of considering local contexts when interpreting LARC utilization patterns. While our study supports the broader trend observed in SSA [5] that married women are more likely to utilize LAC, the DRC’s experience suggests that socio-cultural factors can significantly alter these trends.

Living in middle or rich economic classes increases the odds of LAC utilization by 13% and 16% more than women living in poor households. This interaction was also observed in a similar study conducted by employing SSA DHS data [5]. One plausible explanation is that somewhat wealthy women may have greater access to family planning information, as well as the financial resources to pay for transportation or preventative care if necessary. Our study provides a clearer picture of how economic class impacts access to and utilization of LAC, emphasizing that the disparity is quantifiable.

Women with some form of education were more likely to utilize LACs compared to those with no education. This is a result of the majority of educated women having easier access to data regarding the advantages and disadvantages of utilizing LAC methods [40]. As a result, they are aware of the myths and misconceptions that frequently act as barriers to the application of LACs. Studies conducted in Kenya [41], Ethiopia [6], and Uganda [42] indicate that higher educational attainment is associated with greater contraceptive knowledge and acceptance, reflecting a broader regional trend. The nuanced differences in the impact of education across various contexts highlight the need for tailored interventions that address local barriers to contraceptive utilization while leveraging educational advancements to enhance LAC adoption.

Having a history of pregnancy termination reduced the odds of using LACs by 15% as compared to not having terminated a pregnancy before. This finding was concurrent with the study conducted in Ethiopia [15]. This could be explained by the fact that the people were unable to have enough children since they had previously had abortions. As a result, they can have as many children as they like. In contrast, another study from Angola revealed that women with a history of abortion were more likely to utilize modern family planning compared to women who never had an abortion [43]. This difference suggests that, in some contexts, women with a history of abortion may be more motivated to prevent future unintended pregnancies, resulting in higher LAC adoption. It highlights the need to consider local contexts and healthcare environments when evaluating how abortion history influences contraceptive choices. Tailored interventions addressing the specific needs of these women could enhance LAC utilization.

In addition, having a specific work increased the likelihood of using LACs by 28% compared to respondents without a job. Ethiopian reports corroborated this condition [6,37]. The potential explanation for this could be their employment-related advantage in learning about efficient family planning techniques, such as LACs, which allows them to defy the conventional wisdom that forbids LAC utilization [6]. This suggests that targeted interventions leveraging employment-related resources could boost LAC adoption.

A significant association was observed between fertility preference and LAC utilization. Women having didn’t want to have more children were 1.57 times more likely to utilize LACs, compared with women who wanted other children. This interaction was supported by a certain study conducted in Rwanda [44] and a variety of reports generated from Ethiopia [45,46]. Women who don’t want any more children may be more likely to utilize LACs to delay or stop having children due to their safety, efficacy, and long-term advantages [47,48].

Women who had some media exposure had a 21% higher likelihood of utilizing LACs as a family planning method than women who had no media exposure. This result is consistent with [15,45]. The increased utilization of LACs among women exposed to the media may be attributed to the availability of LACs’ advertisements and programs through the media, which serves as a family planning approach.

The study also showed that reproductive-age women with three to four children and those with five or more children had a similar likelihood of using LACs which is higher utilization compared with those with one to two children. However, having no living children at home decreases the odds of LAC utilization by 87% compared to having at most two living children. It is in line with results from Iran [49], Malawi [13], and Ghana [16]. Having no living children at home might increase the desire to get a child, this condition could hinder the utilization of contraceptives, especially LACs. Moreover, having an increased number of living children may make the women achieve the desired number that women limit further pregnancies using LACs [50,51]. A possible explanation for this finding could be that multiparous women tend to receive family planning education and counseling on contraceptive utilization throughout their pregnancy cycle, thereby increasing their odds of using LACs [52].

According to our research, the utilization of long-acting contraceptives decreased by 10% among women who had visited HFWs within the previous year. The lack of HFWs with the training necessary to provide long-acting contraceptive services or the lack of logistics in the health posts could be the cause of this. Furthermore, the inclusion of female sterilization in our study may have an impact on our findings.

When compared to their respective ones, women who had their first sexual experience after turning 18 and those who had their first child after turning 20 exhibited lower LAC utilization. A Malawian study [53]provides support for this investigation. Given the correlation between the two predictor variables, women in this age range are more likely to be pregnant for the first time and decide against wanting another child within three years, which leads them to choose the LACs approach [54].

The level of community illiteracy is one of the community-level (country) components that has the strongest correlation with the utilization of LACs. Consequently, compared to women from countries with high levels of illiteracy, the frequency of LAC consumption is higher among women from nations with low levels of literacy. This result was in line with other publications’ findings [31,55]. Women who have completed secondary school have a very good chance of controlling their reproductive needs. The likelihood that women will utilize LACs increases with their level of education. This might be because women with higher levels of education are more likely to be able to receive information on contemporary methods of contraception, have a better understanding of these techniques, and be more willing to utilize the services [31,55].

Furthermore, regarding community-level variables, women from wealthy households were less likely to utilize LACs, a finding supported by a study conducted in Nigeria[31].

This contrasts with other research, including our individual-level study, which found that family wealth positively correlates with LAC utilization and that wealthier women utilize LACs more frequently than poorer women [49,56].

Finally, a community with higher media coverage of family planning methods has a higher utilization of LACs(30).

The study’s initial strength was that it was carried out utilizing combined data from a single nationally representative DHS survey performed in East African nations. As a result, there was sufficient power in this high sample size to identify the real impact of the independent variables. Secondly, to get accurate estimates and standard errors, the sample weight was applied throughout the analysis. As a drawback, a causal association cannot be demonstrated because the study employed cross-sectional data. We were unable to access crucial variables including behavioral characteristics, and the caliber of the health services, since we were using secondary data analysis. Additionally, the study did not account for women’s perceptions of LAC utilization and relied on self-reported contraceptive use, which may impact data accuracy.

## Conclusion

We discovered that the 11 Eastern African nations included in this study had comparatively low rates of LAC utilization. Reproductive-age women should be counseled on the advantages of utilizing LACs. This study found that parameters related to the utilization of LACs were related to both individual and country-levels. Consequently, women who are married, come from better-off households financially, are educated, have a job, are not desirous of having children, have been exposed to the media, have had more than three children, and are from nations with lower rates of illiteracy, poverty, and media exposure tend to utilize LAC methods more frequently. However, women who had previously had a pregnancy aborted, been visited by a HFW in the previous year, had their first sexual experience after the age of 18 and had their first child after the age of 20 have a lower utilization of LACs.

### Recommendations

Reproductive-age women in East African nations should be counseled about the advantages of using LACs. The implementation of health promotion programs by governments, policymakers, and stakeholders can raise awareness and increase the need for LAC utilization among reproductive-age women in these nations. Countries in Eastern Africa should give priority to implementing educational programs for reproductive-age women with lower levels of education. Moreover, reproductive-age women who utilize LACs sparingly should be encouraged to participate in intervention programs such as campaigns that highlight the effectiveness of LACs in lowering unwanted pregnancies, maternal mortality, and morbidity.

## Abbreviation

AOR: Adjusted Odds Ratio
DHS: Demographic Health Survey
EDHS: Ethiopian Demographic Health Survey
IUD: Intrauterine Device
HFWs: Health Fieldworkers
LACs: Long-Acting Contraceptives
SSA: Sub-Saharan Africa
Vnull: Variance at the group level from the null model
VC: Variance at the group level from the model with variables included
